# Effects of the spray-drying process using maltodextrin on bioactive compounds and antioxidant activity of the pulp of the tropical fruit açai (*Euterpe oleracea* Mart.)

**DOI:** 10.1016/j.heliyon.2024.e33544

**Published:** 2024-06-25

**Authors:** Valentina Vargas, Sebastian Saldarriaga, Francis S. Sánchez, Liceth N. Cuellar, Gloria M. Paladines

**Affiliations:** Grupo de Investigación en Productos Naturales Amazónicos GIPRONAZ, Universidad de la Amazonia, Calle 17 Diagonal 17-Carrera 3F, Florencia, Colombia

**Keywords:** Spray drying, Response surface methodology, Bioactive compounds, Encapsulation

## Abstract

Aҫai fruit is characterized by the properties of its bioactive compounds; however, this fruit is highly perishable and its compounds are sensitive when exposed to non-optimal environmental factors. Therefore, the objective of this study was to encapsulate the fruit pulp by spray drying to improve the nutritional value and extend the shelf life of the products derived from acai fruit. Maltodextrin was used as a wall material and the process was optimized to obtain the desirable values of the response variables. For this, a central compound design (CCD) was developed to determine the influence of temperature (110–170 °C) and the wall material proportion (5–15 %) on dependent variables: the retention of ascorbic acid, moisture percentage, hygroscopicity, solubility, water activity, and yield. Furthermore, the effects of spray drying on bioactive compounds (AA, TPC, TFC, TA, TCC, GA, CT, and QC) and antioxidant activity (ABTS, DPPH, and ORAC) were evaluated. The maximum design temperature (170 °C) and wall material proportion (15 %) significantly influenced the response variables where encapsulation was applied, with high ascorbic acid retention (96.886 %), low moisture (0.303 %), low hygroscopicity (7.279 g/100 g), low level of water activity (0.255), a water solubility index of 23.206 %, and a high yield of 70.285 %. The bioactive compounds analyzed and the antioxidant capacity presented significant retention values for AA (96.86 %), TPC (65.13 %), TFC (82.09 %), TA (62.46 %), TCC (7.28 %), GA (35.02 %), CT (49.03 %), QC (37.57 %), ABTS (81.24 %), DPPH (75.11 %), and ORAC (15.68 %). Therefore, it is concluded that the powder obtained under these conditions has desirable physical properties, and the drying process preserved a notable retention of bioactive compounds and their antioxidant activities.

## 1 Introduction

Aҫai (*Euterpe oleracea* Mart.) is a tropical fruit native to the Amazon region that has gained significant popularity in recent years because of its pulp, which constitutes 16 % of the fruit composition and holds remarkable potential for health benefits and economic value [1]. From this fruit, various products such as ice cream, energy drinks, cosmetics, and nutritional supplements can be prepared [2,3]. The açai extract has also garnered recognition for its properties attributed to its anthocyanin content, which is responsible for the fruit's distinctive purple hue [4], as well as phenols, flavonoids, and antioxidants, which act as anti-inflammatory, antimicrobial agents [5], and cardiovascular protectors, as demonstrated by Alessandra-Perini et al. [6], in their study where Aҫai extract shows cardioprotective effects in the progress of breast cancer. One of the most important antioxidants present in açaí fruit is ascorbic acid, commonly known as vitamin C, which plays a crucial role within the human body, participating in various physiological processes including collagen synthesis, immune system functionality, and iron absorption [7].

However, the properties of all these compounds in açai fruit can be compromised due to the fruit's perishable nature [8]. Additionally, both ascorbic acid and anthocyanins are unstable compounds susceptible to degradation when exposed to suboptimal temperatures, light, and moisture conditions [9]. Therefore, techniques, such as encapsulation by spray drying, have been developed to mitigate the factors affecting the stability of such compounds [10]. Spray drying is a crucial technique in food processing, particularly for fruits. This process involves the transformation of liquid substances, such as fruit extracts and juices, into solid and easily dispersible particles. The encapsulated particles not only shield these valuable components against degradation caused by exposure to oxygen, light, and moisture, but also prolong the shelf life of the products derived from the fruits [11,12]. Although there are various methods for carrying out the microencapsulation process such as coacervation, freeze-drying, co-crystallization, interfacial polymerization, ionic gelation, and polymer incompatibility, it has been demonstrated that spray drying is the most widely used as it is a fast, cost-effective process with good yields and efficiency rates [4]. Furthermore, this technique preserves organoleptic and functional properties [12] and offers rapid reconstitution of the microencapsulated material in water [13].

In general, the encapsulates obtained from spray-drying processes demonstrate a wide array of characteristics and properties that are influenced by numerous factors. By fine-tuning the process variables to their optimal values, it is feasible to obtain powders with favorable yields, low water activity, minimal moisture content, low hygroscopicity, high solubility, and substantial retention of metabolites and biological activities retention [13]. The efficiency of this process depends on several factors, including the inlet air temperature, feed flow rate, preparation of the feed solution, and proper incorporation of wall materials or encapsulating agents [14]. Currently, a variety of these agents have been developed to accommodate emerging applications. The selection of these materials primarily depends on their properties and compatibility with the bioactive compound, which facilitates the interaction and formation of the encapsulating layer. Maltodextrin is one of the most extensively used encapsulating agents, predominantly owing to its ability to encapsulate compounds that are sensitive to temperature variations, such as vitamins, polyphenols, and some antioxidants. The advantages of this material stem from its low cost and high efficiency. It lacks odor and color, exhibits excellent water solubility, and maintains a low viscosity, even at high concentrations [15]. However, it is important to note that the spray drying technique entails losses attributed to the factors mentioned above, and this process can significantly influence the resulting properties of the powder obtained [[16], [17], [18]].

Currently, studies have explored the encapsulation of açai pulp by spray drying [4,[17], [18], [19], [20], [21]]. Most focus has been on optimizing the retention of compounds, specifically anthocyanins, and evaluating the physical properties of the powder to obtain a higher quality açai powder. However, these studies have not determined the operating conditions to optimize the retention of compounds that are less studied in açai, such as ascorbic acid. Additionally, the effects of the optimized conditions on the retention of phenolic compounds and antioxidant activity have not been explored. Therefore, this work focuses on the retention of these compounds, which are vital given the perishable nature of açai fruit. By optimizing the encapsulation to preserve these metabolites, the aim of this study was to improve the nutritional value and extend the shelf life of açai products, offering significant benefits for consumers and the food industry. The research was performed by modifying the inlet air temperature and the proportion of the wall material to determine the conditions that preserve the highest ascorbic acid content and encapsulation properties. Finally, the bioactive and antioxidant characteristics of both the pulp and the resulting powder were compared to evaluate the effects of the drying process on the previously mentioned properties.

## 2 Materials and methods

### 2.1 Materials and reagents

Ripe aҫai pulp was purchased from Puramazonia ZOMAC SAS (Caquetá, Colombia). Maltodextrin (DE 15–20) was used as the wall material and was procured from Cimpa (Medellín, Colombia). Potassium persulfate (K_2_S_2_O_8_), ascorbic acid, 6-hydroxy-2,5,7,8-tetramethylchroman-2-carboxylic acid (Trolox), and 2,2-diphenyl-1-picrylhydrazyl (DPPH) were obtained from Sigma-Aldrich. Gallic acid and aluminum chloride (AlCl_3_) were obtained from Alfa Aesar. 2,2′-azino-bis (3-ethylbenzothiazoline-6-sulfonic acid) (ABTS), methanol, ethanol, and sodium chloride (NaCl) were obtained from Merck (KGaA, Darmstadt, Germany). All other chemicals, including Folin–Ciocalteu's reagent, anhydrous sodium carbonate (Na_2_CO_3_), sodium nitrite (NaNO_2_), sodium hydroxide, (+)-catechin, acetone, monobasic potassium phosphate, and dibasic potassium phosphate, were obtained from PanReac AppliChem.

### 2.2 Feed solution preparation and the spray-drying process

The pulp was obtained by washing the ripe acai fruits with water, and a pulping machine was used to separate the pulp from the peel and seeds. Water was added during pulping in a 1:0.4 ratio of acai pulp:water, and then the pulp was stored in a Haier Ultra-low temperature freezer (DW-86W100, China) at −40 °C until use. For drying, the pulp was thawed at 4 °C for 12 h and filtered up to 4 times through an ultra-fine nylon mesh (pore diameter of 150 μm) to retain insoluble solids and reduce nozzle clogging. Total soluble solids were measured using a digital refractometer (MA888). For each experiment, 300 mL of filtered pulp and the respective wall material proportion (5–15 % corresponding to the mass ratio) were homogenized using an Ultra-Turrax T-25 (IKA, Germany) at 8000 rpm for 5 min.

The feed solutions were then placed in a Mini Spray dryer (Buchi B-290, Flawil, Switzerland) for spray drying. This was carried out at different inlet air temperatures (110–170 °C) and a constant feed flow rate of 25 % (7.5 mL/min). These variables were defined according to preliminary tests and Tonon et al. [17] and da Costa Valente et al. [4], with some modifications. The operational conditions were chosen based on a study by Pinheiro et al. [22]. The mixture was fed into the main chamber using a peristaltic pump with a two-fluid nozzle atomization system. The inlet air flow was 40 m^3^/h and the compressor air pressure was 0.06 MPa, with a 0.7 mm inner diameter injector nozzle and 100 % suction air flow. The humidity of the environment during the spray drying process was approximately 80 %. The aҫai powders were kept in hermetic bags protected with aluminum and stored in a vacuum desiccator until analysis.

### 2.3 Physical properties of acai powder produced by spray drying

#### 2.3.1 Ascorbic acid encapsulation efficiency (AE)

The ascorbic acid content was determined in triplicate by standard addition using the differential pulse voltammetry (DPV) technique described by Pisoschi et al. [23] with some modifications. A 797 VA Computrace potentiostat (Metrohm, Switzerland) was used with a glassy carbon electrode (GCE) as the working electrode, Ag/AgCl as the reference electrode, and Pt as the auxiliary electrode. For this method, a 1 g/L ascorbic acid standard was used. The measurements were performed in an electrolytic cell which contained 5 mL of phosphate buffer saline (PBS) (100 mM and pH of 2.1) and 100 μL of diluted (1:10) acai extract. Parameters used for the determinations were pulse amplitude of 100 mV and pulse time of 10 ms. Before carrying out the measurements, the working electrode was cleaned on the polishing pad with alumina (Al_2_O_3_) of 0.38 μm. The electrode was washed with deionized water to remove traces of alumina and then sonicated for 1 min in deionized water using an Ultrasonic Cleaner model DK-1000PF (DK SONIC, China)**.** The ascorbic acid content in the acai pulp powder was expressed as encapsulation efficiency according to Equation (1) (Table S10) [24].

(1) $$\text{AE}\left(\%\right)=\frac{C1\;*M_{e}}{C_{p}*M_{p}}*100\%$$

where C1 is the concentration of ascorbic acid in the powder (mg/100 g), C_p_ is the concentration of ascorbic acid in the acai pulp, M_e_ is the amount of powder obtained, and M_p_ is the amount of pulp introduced during feeding.

#### 2.3.2 Encapsulation yield (Y)

The encapsulation yield was determined as the ratio between the mass of dry acai powder obtained after the spray-drying process and the amount of solids in the feed solution (amount of wall material plus soluble solids in acai pulp (9.34 g/100 g)). The encapsulation yield is expressed as a percentage and calculated using Equation (2).

(2) $$Y\left(\%\right)\frac{M_{p}}{M_{e}}*100\%$$

where M_p_ is the mass of the dry acai powder and M_e_ is the amount of solids in the feed solution.

#### 2.3.3 Moisture (M)

Moisture content was gravimetrically determined in triplicate by subjecting 1 g of the sample at 105 °C in a vacuum oven (model OV-11, Jeio Tech Co., Korea) for 5 h, according to the Official Methods of AOAC (method 925.45B). The results are expressed as percentages, calculated using Equation (3).

(3) $$\text{Moisture}\left(\%\right)=\frac{w_{i}-\;w_{f}}{w_{i}}*100\%$$

where w_i_ and w_f_ are the initial and final weights of the sample, respectively.

#### 2.3.4 Water solubility index (WSI)

The water solubility index was determined in triplicate according to the method described by Tonon et al. [18], with some modifications. For each measurement, 1 g of acai powder was diluted in 100 mL of distilled water and magnetically stirred for 15 min. Then, the solution was centrifuged at 3000 rpm (LC-04R OSSALUD, Colombia) for 15 min, and 25 mL of the supernatant was transferred to a Petri dish and dried in a vacuum oven (OV-11, Jeio Tech Co., Korea) at 105 °C for 5 h. WSI was expressed as a percentage and was calculated as the ratio between the mass of the dry supernatant (M_s_) and the initial mass of the powder (M_p_) as described in Equation (4).

(4) $$\text{WSI}\left(\%\right)=\frac{M_{s}}{M_{p}}*100\%$$

#### 2.3.5 Hygroscopicity (Hyg)

Hygroscopicity was determined using the methodology proposed by Cai and Corke [25]. Approximately 1 g of acai powder was placed in a pre-weighed beaker in a desiccator containing saturated sodium chloride (NaCl) solution at room temperature. The powders were reweighed after 7 days. Hygroscopicity was expressed as the mass of absorbed water per 100 g of powder, calculated using Equation (5). This parameter was measured in triplicate.

(5) $$Hyg\;\left(\frac{g}{100\;g}\right)=\frac{W_{f}\;-\;W_{i}}{W_{i}}*100\%$$

where w_i_ and w_f_ are the initial and final weights (after 7 days) of the sample, respectively.

#### 2.3.6 Water activity (a_w_)

Water activity (a_w_) was measured in triplicate using a LabSwift water activity analyzer (Novasina, Germany) at room temperature.

### 2.4 Experimental design and optimization of the drying process

Optimization of the inlet temperature (B) and wall material proportion (A) as independent variables was performed using the Response Surface Methodology (RSM). A central compound design (CCD) was applied according to da Costa Valente et al. [4] and Tonon et al. [17] to determine the number of experiments and study the effects of independent variables on the response variables after the encapsulation of acai pulp by spray drying. The CCD was based on four factorial points (±1 levels), four axial points (one variable at the ±α level and one at the zero level), and four replicates of the central point (level 0), resulting in a total of twelve experiments. The response variables were ascorbic acid encapsulation efficiency (AE), yield (Y), moisture (M), water solubility index (WSI), hygroscopicity (Hyg), and water activity (a_w_). Design-Expert software (version 11) was used for the experimental design.

### 2.5 Characterization of acai pulp and the powder obtained under optimal spray drying conditions

#### 2.5.1 Sample preparation

The pulp (**∼**2 g) and acai powder (0.02–1 g) were separately extracted with 1.8–10 mL of water and vigorously stirred for 10 min in a multi-reax tube shaker (Heidolph, Germany). The extracts were centrifuged using a centrifuge (SL 8R, Thermo Fisher Scientific) at 4500 rpm for 10 min, and the supernatants were filtered. The resulting extracts were used for bioactive compound and antioxidant analyses using a MultiSkan Go plate reader (Thermo Fisher Scientific).

For HPLC-UV analysis, 100 mg of each sample (pulp or powder) was homogenized in 1 mL of methanol:water:formic acid (25:24:1, v/v/v) for 60 min under ultrasonication (Mujigae SD-330HT, Korea) and kept at 4 °C overnight. Then, the extracts were separately centrifuged at 10,000 rpm for 15 min, and the supernatants were filtered through PVDF syringe filters (0.22 μm, 13 mmØ, Análisis Vínicos, Tomelloso, Spain). All extracts were stored at 4 °C until analysis, and all experiments were carried out in triplicate.

#### 2.5.2 Ascorbic acid (AA)

The ascorbic acid content in the extracts was determined following the methodology described in section 2.3.1 and was expressed as ascorbic acid retention (%) according to Equation (1).

#### 2.5.3 Total phenolic content (TPC)

TPC was determined spectrophotometrically using the Folin-Ciocalteu colorimetric method [26], with some modifications. The extract (18 μL) was diluted by adding 124.5 μL of deionized water. Then, 37.5 μL of Folin-Ciocalteu reagent and 120 μL of 7.1 % anhydrous sodium carbonate (Na_2_CO_3_) were added. The extract was kept in the dark at room temperature (24 °C) for 60 min, and the absorbance was read at 760 nm. Gallic acid was used as the standard. The results are expressed as mg of gallic acid equivalent (GAE)/100 g of acai pulp or powder.

#### 2.5.4 Total flavonoid content (TFC)

The TFC was determined by the reaction with aluminum chloride (AlCl_3_). This method was based on that of Woisky and Salatino [27], with some modifications. The mixture consisted of 120 μL of deionized water, 30 μL of the extract, sodium nitrite (NaNO_2_) (9 μL, 5 %) (left for 5 min), and aluminum chloride (AlCl_3_) (9 μL, 10 %) (left for 5 min). Then, 60 μL of 1 M sodium hydroxide (NaOH) was added (left for 15 min), followed by the addition of 72 μL of deionized water. The mixture was left to react in the dark at room temperature for 30 min and the absorbance was measured at 510 nm. (+)-Catechin was used as a standard, and the results were expressed as milligrams of catechin (CAT)/100 g of acai pulp or powder.

#### 2.5.5 Total carotenoid content (TCC)

TCC was quantified according to the method proposed by Neves et al. [28], with some modifications. A volume of 6 mL of acetone (99.5 %, v/v) was added to 2 g of the extract. The samples were vortexed for 15 min and centrifuged at 4500 rpm for 10 min. Finally, the supernatant was separated and its absorbance was read in a quartz cell at 450 nm. ẞ-carotene was used as the standard and the results were expressed as mg of ẞ-carotene/100g of acai pulp or powder.

#### 2.5.6 Total anthocyanins (TA)

The TA content was determined according to the method of Giusti and Wrolstad [29] using the differential pH method. The extracts were diluted with potassium chloride (KCl) buffer (0.025 M, pH 1.0) and sodium acetate (CH_3_CO_2_Na–3H_2_O) buffer solution at pH 4.5 (0.4 M). The absorbance was measured at 510 and 700 nm. The total anthocyanin content was expressed as mg/100 g and anthocyanin retention as %, calculated using Equations (6), (1), respectively.

(6) $$\mathrm{TA}\;\left(\frac{\text{mg}}{100\;g}\right)=\frac{\left(A*\;\text{MW}*\;\text{DF}*\;1000\right)}{\left(\varepsilon *l\right)}$$

where A (absorbance) corresponds to (A_***λ***__510_ – A_***λ***__700_) _pH 1.0_ – (A_***λ***__510_ – A_***λ***__700_) _pH 4.5_, MW is the molecular weight of cyanidin-3-glucoside (449.2 g/mol), DF is the dilution factor used, ***ε*** is the molar absorbance of cyanidin3-glu6coside (26.900 g/mol), and “l” is the cell length (1 cm).

#### 2.5.7 ABTS^•+^ radical scavenging activity (ABTS)

ABTS was determined according to the method proposed by Re et al. [30] with some modifications. The ABTS^•+^ radical was generated by reacting the ABTS stock solution (10 M) with potassium persulfate (2.45 M), and the solution was left to react in the dark at room temperature for 16 h. The ABTS^•+^ radical solution was diluted in phosphate buffer saline (PBS) (0.15 mol/L and pH 7.4) to reach an absorbance of 0.7 at 734 nm. For the reactions, 3 μL of each extract was added to 297 μL of the ABTS^•+^ radical solution and the absorbance was read at 734 nm after 30 min of reaction in the dark. Results were expressed as TEAC values in μmol Trolox/g acai pulp or powder, using a Trolox standard curve [31].

#### 2.5.8 DPPH radical scavenging activity (DPPH)

DPPH was determined according to the method proposed by Neves et al. [28] with some modifications. DPPH stock solution (20 mg/L) was prepared in absolute methanol at 4 °C. The absorbance of the radical was adjusted to 0.3 with methanol at 4 °C. For the reaction, 3 μL of each extract was added to 297 μL of the adjusted DPPH solution and the absorbance was read at 517 nm after 30 min of reaction in the dark at room temperature. The results were expressed as TEAC values in μmol of Trolox/g of acai pulp or powder, using Trolox as the standard**.**

#### 2.5.9 Oxygen radical absorbance capacity (ORAC)

ORAC was determined according to the method described by Ou et al. [32]. For the assay, 2600 μL of fluorescein solution (0.04 μM) (previously prepared in a phosphate buffer (0.075 M) at pH = 7.0) and 100 μL of the sample were mixed in a 4 mL test tube. After 30 min of incubation at 37 °C, 300 μL of 2,2′-azobis(2-methylpropionamidine) dihydrochloride (AAPH) solution (200 mM) was added to initiate the reaction (the AAPH solution was previously prepared in a phosphate buffer solution (0.075 M) at pH = 7.0). Fluorescence measurements were performed at 37 °C using RF-6000 spectrofluorometer (Shimadzu, Kyoto, Japan). Excitation and emission wavelengths were 485 and 520 nm, respectively. The fluorescence was monitored for 60 min. For the blank sample fluorescein solution (0.04 M) and 100 μL of phosphate buffer were used. The results were expressed as TEAC values in μmol of Trolox/g of acai pulp or powder, using Trolox as the standard, and were calculated according to Equation (S3).

#### 2.5.10 HPLC-UV analysis of phenolic compounds

HPLC-UV analysis was performed according to the method described by Radeka et al. [33] with some modifications. The identification was performed in a HPLC-UV (Shimadzu LC-2010A HT), equipped with a Luna C18 column (250 × 4.6 mm, 5 μm; Phenomenex, Macclesfield, UK). The injection volume was 30 μL, and the chromatograms were recorded at 280 and 320 nm. The phenolic compounds were identified based on their UV/Vis spectra and retention times. The compounds were quantified by establishing calibration curves using gallic acid (GA) at 280 nm for phenolic compounds, catechin (CT) at 280 nm for tannins, and quercetin (QC) at 280 nm for flavonoids. The mobile phase consisted of water:formic acid solution (99:1, v/v) (solvent A) and acetonitrile (solvent B). The flow was 0.8 mL/min in a linear gradient, starting at 6 % B for 5 min, 24 % B for 18 min, which was maintained for 7 min, 60 % B for 30 min, and 95 % B for 40 min. All samples were extracted in triplicate and injected three times. All standard curves were run in the range of 0.015–1 mM (stock solution), and the LOQ (limit of quantification) was close to 0.01 mM in each case whereas the R^2^ was higher than 0.99. The results are expressed as milligram equivalents of the standard/100 g of dry weight.

#### 2.5.11 Scanning electron microscopy (SEM)

The morphology of the optimized powder was studied using a Tescan Lyra 3 scanning electron microscope (SEM) (TESCAN, Czech Republic). A voltage of 15 kV was used with a vacuum of 0.009 Pa. The powder was coated with gold nanoparticles using a desk V thin-film deposition solution (Denton Vacuum, Moorestown, NJ, USA) to increase the electrical conductivity of its surface [24]. The particle size was determined using ImageJ software (version 1.8.0).

### 2.6 Statistical analysis

The optimization of the drying process was performed using the Design Expert software (version 11), and analysis of variance (ANOVA) at a 95 % level of confidence was performed using Response Surface Methodology (RSM) to determine the statistical significance of each model. Coefficients of determination (R^2^) and p-values were used to validate the model. Pearson correlation analysis was performed using InfoStat software (version 2020). The values of experiments were expressed as means ± standard deviation. SigmaPlot software (version 15.0) was used to perform the statistical analysis of the data of bioactive compounds and antioxidant activity.

## 3 Results and discussion

### 3.1 Physicochemical properties of acai powder produced by spray drying

The optimization process was performed for the inlet temperature and wall material proportion, primarily to ensure the highest ascorbic acid encapsulation efficiency, as well as high yields, low moisture, low hygroscopicity, low water activity, and high solubility. Table 1 shows the influence of the independent variables (inlet temperature (B) and wall material proportion (A)) on the physicochemical properties of the obtained acai powders, whereas Table 2 shows the ANOVA results for ascorbic acid encapsulation efficiency.

**Table 1:** Physicochemical properties of acai powder.

Experiment	**Independent variables**		Response variables					
	B (°C)	A (%)	AE	Y	M	WSI	Hyg	a_w_
**1**	110	5	17.92 ± 2.31	61.94	2.33 ± 0.05	20.11 ± 0.09	20.67 ± 0.26	0.44 ± 0.02
**2**	170	5	84.72 ± 1.16	75.58	0.20 ± 0.09	19.81 ± 0.19	23.15 ± 0.10	0.23 ± 0.01
**3**	140	10	74.10 ± 1.78	59.10	0.49 ± 0.29	22.40 ± 0.25	19.27 ± 0.34	0.33 ± 0.04
**4**	110	15	79.54 ± 7.04	77.17	2.68 ± 0.07	22.97 ± 0.33	4.41 ± 0.14	0.40 ± 0.03
**5**	170	15	98.90 ± 1.00	74.96	0.28 ± 0.11	23.39 ± 0.66	6.78 ± 0.08	0.28 ± 0.01
**6**	140	7.5	82.31 ± 0.52	72.84	0.98 ± 0.32	21.76 ± 0.24	19.28 ± 0.26	0.37 ± 0.01
**7**	140	12.5	88.93 ± 1.66	73.96	1.38 ± 0.32	22.70 ± 1.24	8.42 ± 0.27	0.35 ± 0.02
**8**	125	10	33.16 ± 6.21	52.12	1.85 ± 0.44	22.46 ± 0.75	10.30 ± 0.61	0.37 ± 0.02
**9**	155	10	86.83 ± 4.76	80.28	0.35 ± 0.05	21.78 ± 0.04	10.63 ± 0.30	0.26 ± 0.01
**10**	140	10	71.61 ± 9.72	57.16	0.81 ± 0.04	22.02 ± 0.40	16.18 ± 0.75	0.35 ± 0.01
**11**	140	10	59.76 ± 4.58	59.86	0.75 ± 0.12	22.03 ± 0.17	18.64 ± 0.12	0.36 ± 0.01
**12**	140	10	64.10 ± 3.91	57.73	0.54 ± 0.49	22.09 ± 0.20	16.54 ± 0.36	0.35 ± 0.02

Values are means ± standard deviation. B, inlet temperature; A, wall material proportion; AE, ascorbic acid encapsulation efficiency (%); Y, yield (%); M, moisture (%); WSI, water solubility index (%); Hyg, hygroscopicity (g/100 g); a_w_, water activity.

**Table 2:** Results of analysis of variance (ANOVA) for ascorbic acid encapsulation efficiency of acai powder as affected by the inlet temperature and the proportion of the wall material.

Source	Effect	F-value	P-value		R^2^	Adjusted R^2^	Predicted R^2^
**Model**		9.30	0.0055*	Significant	0.77	0.6936	0.5241
**A**	17.58	8.10	0.0216*				
**B**	25.11	16.52	0.0036*				
**AB**	−11.86	3.28	0.1079				
**Lack of Fit**		5.65	0.0924	Not significant			

A, wall material proportion; B, temperature.

* Significant value (p < 0.05).

R^2^, coefficient of determination.

The ascorbic acid encapsulation efficiency (AE) varied from 17.92 % to 98.90 % (Table 1), indicating a high efficiency in the drying process. As shown in Table 2, the AE was significantly affected by the wall material proportion (A) and inlet temperature (B) (p < 0.05). The positive effect of the inlet air temperature, shown in Table 2, indicates that the retention of ascorbic acid was favored by an increase in this variable. In this case, the highest AE was obtained at 170 °C and 15 % of wall material with an AE of 98.90 %. This value was similar to those reported by Gadelha et al. [53] who obtained ascorbic acid retentions of 96.8 % in the encapsulation of acerola at 170 °C. Similarly, Pino et al. [34] reported vitamin retentions of 81 % at 170 °C in encapsulated orange. This was unexpected, as vitamin C is thermolabile and tends to degrade rapidly when exposed to high temperatures [35]. However, in the spray drying process, the air temperature was proportional to the drying rate of the microcapsules. Therefore, the obtained results can be attributed to the rapid formation of a microencapsulated semipermeable membrane at high drying temperatures, resulting in a shorter residence time of the drops in the drying chamber, protecting the bioactive compounds and favoring their retention [34]. On the other hand, AE was observed to increase as A increased, showing a positive effect between them, which can be explained by the protective effect of maltodextrin, which is responsible for protecting the active ingredient from oxidation and its resistance to high temperatures [35].

The ANOVA (Table 2) demonstrated that the fitted model for AE was statistically significant (p < 0.05) with a non-significant lack of fit. The R^2^ was 0.7771, indicating that the model explained 77 % of the variation in the observed data. The regression model for ascorbic acid encapsulation efficiency is given by Equation (7). The equation shows a two-factor interaction effect generated from the factors affecting AE.

(7) $$\mathrm{AE}\left(\%\right)=-\;192.85080+14.58290A+1.62749B-0.079050AB^{*}$$

where A corresponds to the wall material proportion and B corresponds to the inlet temperature.

**Fig. 1:**
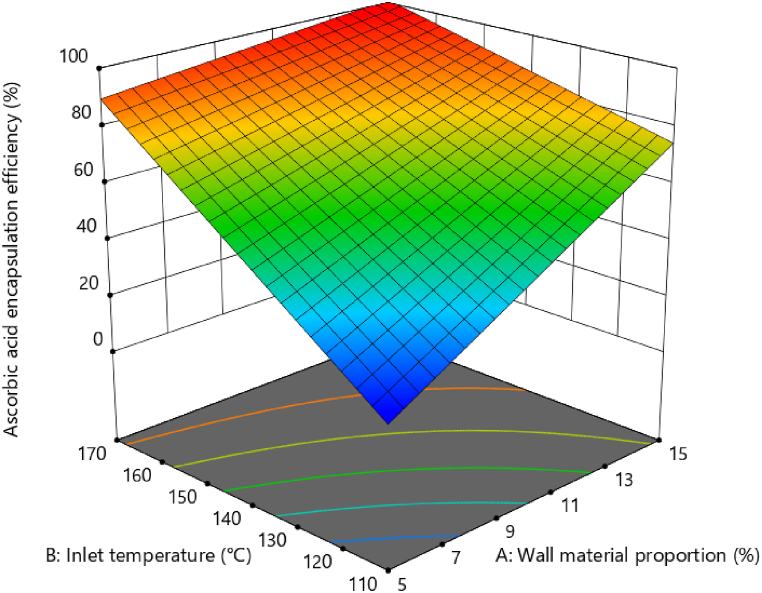
Response surface for the ascorbic acid encapsulation efficiency as a function of temperature and the wall material proportion.

The 2FI model proved to be the most suitable for explaining the effects of the independent variables on AE and also demonstrated a better fit to the experimental data than the quadratic and linear models, generating a response surface (Fig. 1). Based on the surfaces, it can be confirmed that higher retentions occur at high temperatures, and all proportions of the wall material have a significant effect on these values. However, an optimum is obtained at 170 °C and 15 % maltodextrin, with a retention of 98.90 %.

The values of all the response variables were consistent with obtaining a powder with favorable properties and high stability. The yields of encapsulated acai obtained under various conditions ranged from 52.2 % to 80.3 % (Table 1). These yields were notably higher compared to those achieved by Tonon et al. [4,17], where the maximum percentage reached 55.6 % and 55.9 %, respectively, in the encapsulation of aҫai pulp. The yield depends on several factors, including the inlet air temperature and the wall material. In this study, an optimum point was found at 155 °C and 10 % maltodextrin, resulting in a yield of 88.28 %. Similarly, high yields of 74.9 %, 75.5 %, and 77.17 % were obtained when the maximum and minimum factors of the experimental design positively influenced the response variables. High temperatures favor the efficiency of heat and mass transfer, increase the evaporation rate, and therefore reduce the likelihood of particles adhering to the drying chamber, leading to a loss in the mass of the encapsulated material [36]. A previous study [37] also showed similar findings in the drying process of Sirih, where they obtained yield percentages ranging from 4.57 % to 11.24 % at temperatures of 198.11 °C and 230.86 °C. Another study [17] also showed an increase in yield percentage for the encapsulated acai pulp, ranging from 34.39 % to 55.66 % at temperatures of 150 °C and 202 °C.

However, increasing the concentration of the wall material also favors yields in such processes. Using small amounts of maltodextrin, the feed liquid may adhere to the drying chamber because of the sticky behavior of the extracts with high sugar content (glucose, fructose, and sucrose). However, using higher amounts of wall material increases the glass transition temperature, and thus reduces this sticky behavior [38]. Giusti and Wrolstad [39] reported similar findings, obtaining increases in the yield percentages of gelatin encapsulates of 19.32 %, 23.45 %, 27.44 %, and 35.64 % at gelatin:maltodextrin (w/w) ratios of 1:0, 2:1, 1:1, and 1:2, respectively. Germaine et al. [40] also observed the same behavior when evaluating the effect of maltodextrin concentration on the physicochemical properties of kuini powder, with an increase in the yield percentage from 24.61 % to 29.27 % when using 5 % and 20 % maltodextrin. Considering the high yield percentages compared with other studies, the model that best fit the results of this variable was selected. However, as shown in Table S1, these differences were not statistically significant (p > 0.05). This indicates that none of them were able to predict the variation in the experimental data; therefore, this response variable was not included in the optimization process.

**Fig. 2:**
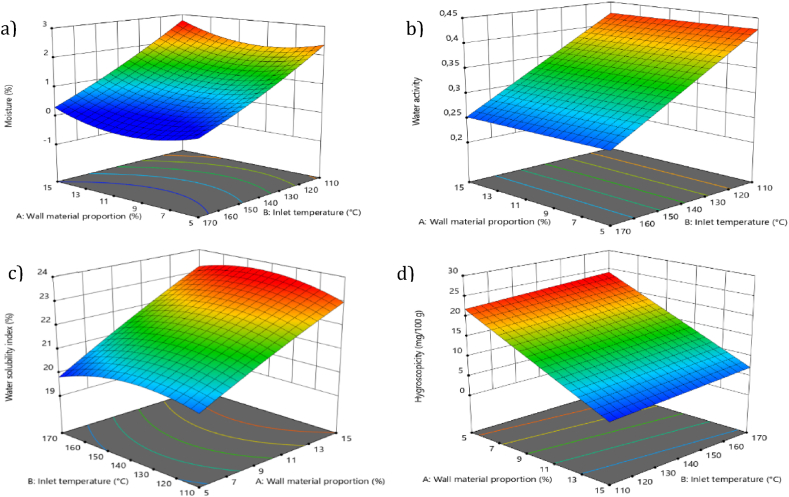
Response surface in terms of temperature and maltodextrin proportion on a) moisture, b) water activity, c) water solubility index, and d) hygroscopicity.

Setting the moisture percentage as a response variable is important, because it ensures the efficiency of the drying process and reduces the moisture content of the encapsulation. This guarantees prolonged and safe storage [16]. The moisture content of açaí pulp powders varied from 0.199 % to 2.68 % (Table 1), similar to values reported by Tonon et al. [17] for açai pulp spray drying but lower than that reported by Fujita et al. [41], which reported a moisture content of 3 % in camu-camu powders. In this case, maintaining low moisture percentages is desirable because percentages below 5 % ensure the efficiency of the drying process, which reduces the moisture content of the powder and, in turn, ensures prolonged and safe storage [42]. According to Table 3, the inlet temperature was the most influential factor on the response variable, exhibiting a negative and desirable effect. Higher temperatures favor products with lower moisture percentages, mainly because a higher inlet air temperature and heat transfer to the particle are activated, thereby accelerating the evaporation of water from the encapsulated material [36]. Similar results were observed in an experiment performed on acai powders, in which a decrease in moisture content from 2.23 % to 1.61 % was reported when the temperature increased from 140 to 200 °C [19]. Likewise, another study [43] reported a decrease in the moisture percentage in the spray drying of watermelon, decreasing from 2.09 % to 1.43 % with an increase in temperature from 120 to 150 °C. The model that best fit the variability of the data was the first-order or linear model, with p < 0.05. However, the quadratic model was selected because of the higher significance of the temperature factor (Table S2), generating a response surface (Fig. 2a) where high temperatures favor a decrease in the percentage of moisture, obtaining an optimum point at 170 °C and a wall material proportion of 5 %, resulting in a moisture level of 0.199 %. ANOVA (Table S3) demonstrated that the adjusted model for moisture percentage was significant, with a p-value <0.05 and a non-significant fit. The coefficient of determination (R^2^) was 0.9306, indicating that the linear model can predict 93 % of the variation in the observed data. The regression model for moisture is given by Equation (8). The equation shows the quadratic effect generated by these factors on moisture.

(8) $$M\left(\%\right)=9.75338-0.260027A-0.071516B-0.000450\text{AB}+0.017544A^{2}+0.000132\;B^{2}$$

where A corresponds to the wall material proportion and B corresponds to the inlet temperature.

**Table 3:** Effects and the degree of significance of the factors in the model applied to the moisture percentage.

Factor	Effect	Standard error	p-value
**A**	0.1395	0.1397	0.3566
**B**	−1.18	0.1397	0.0002*
**AB**	−0.0674	0.1482	0.6649
**A**^**2**^	0.4386	0.6004	0.4926
**B**^**2**^	0.1184	0.6004	0.8502

**A,** wall material proportion (%); **B,** inlet temperature (°C); * significant variables (p < 0.05).

Similarly, water activity demonstrated favorable values, ranging from 0.23 to 0.44 (Table 1), indicating a limited water availability for the growth of microorganisms and promoting stability [16]. Low water activity values were also reported by Manickavasagan et al. [44] for date powder (0.28 at 170 °C with maltodextrin as wall material).

Meanwhile, a previous [45] obtained even lower values, reaching 0.028, when the encapsulation of beet juice was optimized. The results obtained were lower than those reported by Stoll [46], who achieved water activity values of 0.99 in spray drying of açai pulp. The inlet temperature was the most influential factor in the response variable (Table 4), exhibiting a negative and desirable effect, as higher temperatures favor products with low water activity. This behavior is similar to that observed for moisture, where high temperatures facilitate the evaporation of water from the encapsulates owing to the heat transfer process [22]. As shown in Table S4, both linear and quadratic models were significant (p < 0.05). However, the linear model was selected as it best fitted the ANOVA, generating a response surface (Fig. 2b). ANOVA (Table S5) demonstrated that the adjusted model for water activity was significant, with a p-value <0.05, and a non-significant fit. The coefficient of determination was 0.8760, indicating that the linear model can predict 87 % of the variation in the observed data. The regression model for the water activity is given by Equation (9). The equation shows a linear effect generated by these factors on the water activity.

(9) $$a_{w}=0.746019-0.000244A-0.002881B$$

where A corresponds to the wall material proportion and B corresponds to the inlet temperature.

**Table 4:** Effects and the degree of significance of the model factors applied on water activity.

Factor	Effect	Standard error	p-value
**A**	−0.0012	0.0108	0.9127
**B**	−0.0864	0.0108	<0.0001*

**A,** wall material proportion (%); **B**, inlet temperature (°C); * significant variables (p < 0.05).

Water solubility index (WSI) is a crucial parameter in these processes, and values above 80 % are expected [47]. In this study, solubility values ranged from 19.8 % to 23.4 % (Table 1), lower than those reported by Jafari et al. [47], who achieved a maximum percentage of 95 % solubility in pomegranate encapsulation. Similarly, a previous study [24] achieved 90 % solubility for Cupuassu encapsulation using spray drying. According to Table 5, the proportion of the wall material had the greatest influence on the response variable, showing a positive and desirable effect, indicating that an increase in this variable favors the production of powders with higher WSI. This is primarily because of the high solubilizing capacity of the wall material in water [48], which is attributed to the high polarity of the hydroxyl (OH) groups in the molecule [22]. However, the low percentages of solubility in this study could be attributed to the oily phase in açaí pulp due to the presence of characteristic fatty acids, with a high lipid content (48 %) [49], which impeded greater dissolution of the powder in water. As shown in Table S6, both linear and quadratic models were significant (p < 0.05). However, when comparing them through the addition of terms, the quadratic model was selected because it better fitted the data, generating a response surface (Fig. 2c). This confirms what was established by the model, where high proportions of the wall material favor an increase in the solubility percentage, obtaining an optimum point at 170 °C with the maximum maltodextrin proportion of 15 %, resulting in a solubility of 23.39 %. ANOVA (Table S7) demonstrated that the adjusted model for WSI was significant, with a p-value of <0.05 and a non-significant fit. The coefficient of determination was 0.9553, indicating that the quadratic model can predict 95 % of the variation in the observed data. The regression model for the water solubility index is given by Equation (10). The equation shows a quadratic effect generated from the factors on the WSI.

(10) $$\text{WSI}\left(\%\right)=9.39504+0.211134A+0.147376B+0.001210AB-0.003688A^{2}-0.000575B^{2}$$

where A corresponds to the wall material proportion and B corresponds to the inlet temperature.

**Table 5:** Effects and the degree of significance of the factors in the model applied to WSI.

Factor	Effect	Standard error	p-value
**A**	1.53	0.1419	<0.0001*
**B**	−0.0486	0.1419	0.7451
**AB**	0.1816	0.1505	0.2732
**A**^**2**^	−0.0922	0.6100	0.8848
**B**^**2**^	−0.5178	0.6100	0.4285

**A,** wall material proportion (%); **B**, inlet temperature (°C); * significant variables (p < 0.05).

Powder hygroscopicity ranged from 4.41 to 23.15 g/100 g (Table 1), similar to the values reported by Tan [50] in pineapple encapsulation using spray drying, and consistent with the report by da Costa Valente et al. [4] on açaí pulp spray drying. The wall material had the greatest influence on the response variable (Table 6), showing a negative and desirable effect, indicating that increasing the proportion of maltodextrin resulted in powders with low hygroscopicity. This behavior occurs because maltodextrin has low hygroscopicity, which reduces its ability to absorb moisture from the surrounding environment into the powder. This wall material is widely used because its high molecular weight produces powders with high glass transition temperatures (Tg), which minimizes potential structural changes and ensures the stability of the encapsulated material [51]. These low values are favorable because they determine the water absorption capacity of the encapsulates [48]. Considering the models proposed in Table S8, a first-order or linear model was selected, with p < 0.05, which was the best fit according to ANOVA, generating a response surface (Fig. 2d). This confirms what was established by the model, where high proportions of maltodextrin favor low hygroscopicity contents, obtaining an optimum point at 110 °C with a wall material proportion of 15 %, resulting in a minimum hygroscopicity of 4.4146 g/100 g. ANOVA (Table S9) demonstrated that the adjusted model for hygroscopicity was significant, with a p-value <0.05 and a non-significant fit. The coefficient of determination was 0.7916, indicating that the quadratic model is capable of predicting 79 % of the variation in the observed data. The regression model for hygroscopicity is given in Equation (11). The equation shows the linear effect of these factors on moisture content.

(11) Hyg(g/100g)=26.25455−1.69143A+0.037020B

where A corresponds to the wall material proportion and B corresponds to the inlet temperature.

**Table 6:** Effects and the degree of significance of the factors in the model applied to hygroscopicity.

Factor	Effect	Standard error	p-value
**A**	−8.46	1.46	0.0003*
**B**	1.11	1.46	0.4659

**A,** wall material proportion (%); **B**, inlet temperature (°C); * significant variables (p < 0.05).

All response variable results were fitted to linear and quadratic models with predictive capacities for variability of 93 %, 95 %, 79 %, and 87 % for moisture, WSI, hygroscopicity, and water activity, respectively. Regarding the yield, the models were not significant, implying an inability to predict data variability; hence, this response variable was omitted from the optimization process (Table 7). One of the solutions derived from the model, with a wall material proportion of 15 % and an inlet temperature of 170 °C, was chosen to validate the optimal conditions. The predicted value of the ascorbic acid encapsulation efficiency was 99.7 %, and the experimental results are shown in Table 7, where a comparison between the predicted value and the value obtained, indicating that the values of the acai powders obtained at the optimal point were consistent with those predicted because the errors were under 5 %.

**Table 7:** Validation of the optimal conditions predicted by the model (B: 170 °C; A: 15 %).

Response	Predicted value	Experimental value	Error (%)
**AE**	99.7	96.886 ± 2.66	2.82
**M**	0.298	0.303 ± 0.01	1.73
**WSI**	23.048	23.206 ± 0.16	0.69
**Hyg**	7.176	7.279 ± 0.65	1.43
**a**_**w**_	0.255	0.255 ± 0.01	0.08
**Y**	–	70.285 ± 0.51	–

Values are means ± standard deviation. A, wall material proportion; B, inlet temperature; AE, ascorbic acid encapsulation efficiency (%); M, moisture (%); WSI, water solubility index (%); Hyg, hygroscopicity (g/100 g); a_w_, water activity; Y, yield (%).

### 3.2 Characterization of acai pulp and the powder obtained under optimal spray-drying conditions

**Fig. 3:**
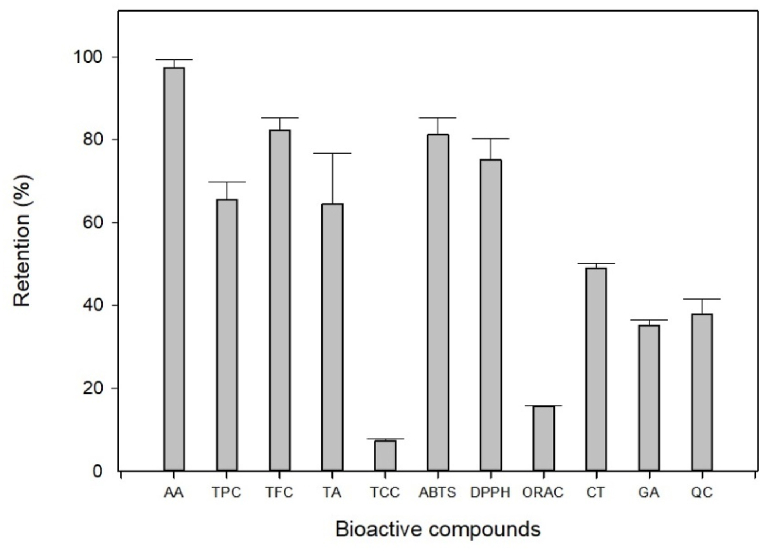
Retention of bioactive compounds after the spray-drying process of aҫai pulp. Error bars correspond to standard deviations of the mean. **AA**, ascorbic acid; **TPC**, total phenolic content; **TFC**, total flavonoid content; **TA,** total anthocyanin; **TCC**, total carotenoid content; **DPPH,** DPPH radical scavenging activity; **ABTS,** ABTS^•+^ radical scavenging activity; **ORAC**, oxygen radical absorbance capacity; **CT,** catechin; **GA**, gallic acid; **QC**, quercetin.

The results of determining the bioactive compounds in açai pulp and its powder obtained through spray drying are presented in Table 8. Equation (1) was used to express these results as retention percentages after drying (Fig. 3). The content of ascorbic acid (AA) exhibited the highest retention percentage (96.35 %) due to the optimization specifically tailored to this compound. This value exceeded that reported by Pino et al. [34] for orange powder obtained through spray drying (89.6 %), as well as that reported by Pinheiro et al. [22] who recorded ascorbic acid retention values between 48.8 % and 89.5 %. This outcome was enhanced by the elevated temperatures at which the drying process was performed, which accelerated the process and reduced contact time with the feed solution in the drying chamber, thereby promoting vitamin retention [34]. The choice of the wall material also played a role in this result, with maltodextrin's resistance to high temperatures contributing to a protective effect that safeguards the active ingredient [52]. This behavior was similar to those reported by Gadelha et al. [53], who achieved 96.8 % retention of ascorbic acid for acerola encapsulation at a temperature of 170 °C.

**Tabla 8:** Bioactive compound contents in acai pulp and powder.

Sample	AA	TPC	TFC	TA	TCC	ABTS	DPPH	ORAC	CT	GA	QC
**Pulp**	32.00 ± 1.01	6239.76 ± 862.89	1180.16 ± 53.58	31.07 ± 6.63	378.90 ± 24.85	1727.88 ± 27.72	32.71 ± 1.02	915.45 ± 0.69	3.22 ± 0.13	32.57 ± 1.23	5.18 ± 0.51
**Powder**	244.40 ± 2.66	34003.99 ± 3172.54	8106.88 ± 132.42	162.40 ± 9.24	230.81 ± 6.80	11745.53 ± 389.44	205.54 ± 6.52	1201.34 ± 2.59	13.22 ± 0.40	95.44 ± 0.68	16.29 ± 0.59

**AA**, ascorbic acid (mg. 100 g-1); **TPC**, total phenolic content (mg GAEa. 100 g-1); **TFC**, total flavonoid content (mg CTb. 100 g-1); **TCC**, total carotenoid content (mg β-Catc. 100 g-1); **TA**, total anthocyanin (mg. 100 g-1); **ABTS**, ABTS•+ radical scavenging activity (μmol TE/g); **DPPH**, DPPH radical scavenging activity (μmol TE/g); **ORAC**, oxygen radical absorbance capacity (μmol TE/g); **CT**, catechin (mg. 100 g-1); **GA**, gallic acid (mg. 100 g-1); **QC**, quercetin (mg. 100 g-1).

a Gallic acid equivalents.

b Catechin.

c β-carotene.

Although the optimization did not focus on all the bioactive compounds in açai fruit, the content of total phenols (TPC) and their flavonoid-type derivates (TFC) remained relatively high even after the spray-drying process, with retentions of 65.12 % and 82.09 %, respectively (Fig. 3). This study demonstrated that elevated temperatures, together with substantial amounts of the wall material, favor greater retention, indicating that spray drying does not severely affect the content of this secondary metabolite. It should be noted that the retention of TPC was comparable to that reported by Pisoschi et al. [24], who achieved a retention of 71.09 % for the encapsulation of the pulp of the Amazonian cupuassu fruit.

For the percentage of anthocyanin retention (TA), a value of 62.45 % was reached, which is lower than that reported by Tonon et al. [17] and da Costa Valente et al. [4], who achieved maximum retentions of 86.01 % and 83.88 %, respectively, for the encapsulation of açaí pulp. This difference may be associated with the high drying temperatures used in the present study and the thermolabile nature of the anthocyanins, which favors possible degradation [54]. Moreover, this result may be due to the type of maltodextrin used, since maltodextrin with different dextrose equivalents (DE) significantly affects the retention of anthocyanins in the encapsulation, as was established in the encapsulation of jussara pulp by spray drying, obtaining values of 88.1 %, 97.4 %, and 98.9 % for maltodextrin with SD of 10, 20, and 30, respectively. In this case, maltodextrin with a DE between 15 and 20 was used, which is considered low, taking into account that maltodextrin (with a dextrose equivalent ≥20) presents a greater degree of hydrolysis and gives rise to the formation of particles that promote pigment conservation.

On the contrary, the content of carotenoids significantly decreased post-drying, reaching a minimum retention of 7.27 %. This behavior contrasts with that of the other bioactive compounds (TPC, TFC, and TA). This outcome can be mainly attributed to the liposoluble nature of carotenoids [55], and the filtration process performed before spray drying likely led to their retention in the ultrafine nylon mesh. Furthermore, carotenoids are prone to oxidation reactions during food processing involving thermal treatments such as spray drying. Consequently, their biological activities were diminished [56]. Similarly, they tend to be sensitive to light because of their unsaturated nature, which allows for their degradation during storage. The wall material also significantly influenced their retention. Rascon et al. [57] demonstrated that the retention of carotenoids in paprika is higher in powders prepared with soy protein than in those prepared with gum arabic.

The antioxidant capacity of açaí pulp and the powder obtained through spray drying were assessed using three methods (ABTS, DPPH, and ORAC), as presented in Table 8, with their respective retentions shown in Fig. 3. Following the encapsulation process, açai demonstrated a significant retention of its antioxidant capacity. The highest retention was associated with the ABTS activity, recording a value of 81.24 %, similar to that reported by Akbarbaglu et al. [58] who achieved 85.62 % retention in flaxseed protein hydrolysates. Likewise, the DPPH radical scavenging activity showed robust performance, achieving a value of 70.11 %, comparable to the findings of a previous study [58] that reported a value of 68.93 %. After spray drying, the activities were notably higher than those of the non-dried sample. The oxidation of polyphenols observed during spray drying led to enhanced capture activity of the samples. In accordance with a previous report [59], partially oxidized polyphenols can significantly enhance radical scavenging efficacy, particularly compared to polyphenols that have not undergone oxidation.

On the other hand, the oxygen radical absorbance capacity (ORAC) showed the lowest retention (15.68 %) (Fig. 3), indicating that the drying process negatively affected this variable. In a study performed by López-Vidaña et al. [60], the ORAC values during the drying process of mortiño varied when three different temperatures were employed, with the highest antioxidant capacity retention observed at 40 °C (54 %), followed by 60 °C (47 %), and finally 50 °C (20 %). The high temperatures used in this study may have caused the decomposition of the antioxidants, thereby reducing their ability to effectively neutralize free radicals and oxidative damage, specifically oxygen radicals. This was confirmed by Lemus-Mondaca et al. [61], who evaluated the effect of the drying temperature on the free radical scavenging activity of ORAC and DPPH in dried Stevia leaves. They reported that samples dried at different temperatures (30–80 °C) did not show significant differences in DPPH free radical scavenging activity. However, for ORAC assays, there was a variation at different temperatures, with the maximum value reached at 40 °C.

Phenolic compounds were identified and quantified in açaí pulp and its powder using HPLC. In this case, gallic acid was identified as a phenolic compound, whereas quercetin and catechin were identified as flavonoids, with retentions of 35.02 %, 37.56 %, and 49.03 %, respectively. This behavior is consistent with the findings of Akther et al. [56], who showed that phenolic compounds exhibited lower retention factors, indicating a loss after the drying process, possibly due to oxidation [62]. Notably, the highest retention was achieved for catechins, which benefited from drying. This enhancement was attributed to the isomerization and hydrolysis interconversion of phenolic compounds and their formation from catechin conjugates [63].

In addition, a Pearson correlation matrix was used to compare the relationships between the antioxidant activity and bioactive compounds (Table 9). According to the results obtained, the correlations were highly significant (p < 0.0001). Phenolic compounds, flavonoids, anthocyanins, and ascorbic acid showed strong positive correlations with the radicals (ABTS, DPPH, and ORAC) (r = 0.98–1.00). Therefore, these compounds are responsible for the antioxidant activity of the acai pulp. Hanula et al. [64] also reported a positive correlation (p < 0.001) between phenol and flavonoid content and antioxidant activity using DPPH and ABTS radicals in microwave and ultrasound extraction methods for acai berries. Similarly, another study [65] reported a high correlation between anthocyanins and antioxidant activity in the ABTS and DPPH assays, with correlation coefficients of r = 0.995 and r = 0.992, respectively. On the other hand, as shown in Table 9, the carotenoid content displayed a negative correlation with antioxidant activity, similar to what was reported by Leme et al. [66], who obtained correlation coefficients of −0.80 and −0.72 for DPPH and ABTS, respectively. Considering these results, it can be concluded that the positive correlations favored high retention of phenolic compounds and antioxidant activity, as shown in Fig. 3.

**Table 9:** Pearson correlation matrix for antioxidant activity and bioactive compounds.

	TPC	TFC	DPPH	ABTS	TCC	TA	ORAC	AA	CT	GA	QC
**TPC**	1.00										
**TFC**	0.99	1.00									
**DPPH**	0.98	0.99	1.00								
**ABTS**	0.98	1.00	0.99	1.00							
**TCC**	−0.97	−0.98	−0.98	−0.98	1.00						
**TA**	0.99	0.99	0.98	0.99	−0.98	1.00					
**ORAC**	0.99	1.00	1.00	1.00	−0.98	0.99	1.00				
**AA**	0.99	1.00	1.00	0.99	−0.98	0.99	1.00	1.00			
**CT**	0.99	1.00	0.99	1.00	−0.98	1.00	1.00	1.00	1.00		
**GA**	0.99	1.00	1.00	1.00	−0.98	0.99	1.00	1.00	1.00	1.00	
**QC**	0.99	1.00	0.99	1.00	−0.98	0.99	1.00	0.99	1.00	1.00	1.00

**AA**, ascorbic acid; **TPC**, total phenolic content; **TFC**, total flavonoid content; **TA,** total anthocyanin; **TCC**, total carotenoid content; **DPPH,** DPPH radical scavenging activity; **ABTS,** ABTS^•+^ radical scavenging activity; **ORAC**, oxygen radical absorbance capacity; **CT,** catechin; **GA**, gallic acid; **QC**, Quercetin.

All correlation coefficient presented p < 0.0001.

This study highlights the complex interplay between factors that influence the retention of bioactive compounds during spray drying. While some compounds such as ascorbic acid and flavonoids were well preserved, others such as carotenoids and ORAC exhibited lower retentions. The choice of drying parameters, wall materials, and the nature of the compounds themselves all play critical roles in determining their retention levels. This encapsulation enhanced the stability of acai, enabling its versatile use in various food and beverage products, while maintaining its potent antioxidant capacity. The consumption and commercialization of the powdered fruit can be expanded by using it as an ingredient in deserts, dietary bars, nutritional supplements, ice cream, and yogurt [67]. Moreover, controlled-release options facilitate optimized bioavailability and health benefits, making encapsulated acai a versatile and practical solution for promoting the overall well-being and developing functional foods, dietary supplements, and nutraceuticals.

This study was compared with that reported by de Jesus Silva [68] in which a protein isolate was used to encapsulate açaí. Although this encapsulant improved the nutritional quality of the final product owing to its protein value, it was observed that the yield values were lower, with approximately 45 %. Furthermore, this study demonstrated significant retention of bioactive compounds (such as anthocyanins, flavonoids, and ascorbic acid) and antioxidant activity (ABTS and DPPH), with notable values ensuring the stability and quality of the final product. Thus, the obtained values highlight the efficiency of the spray-drying process in preserving the beneficial compounds of açai.

### 3.3 Scanning electron microscopy (SEM)

**Fig. 4:**
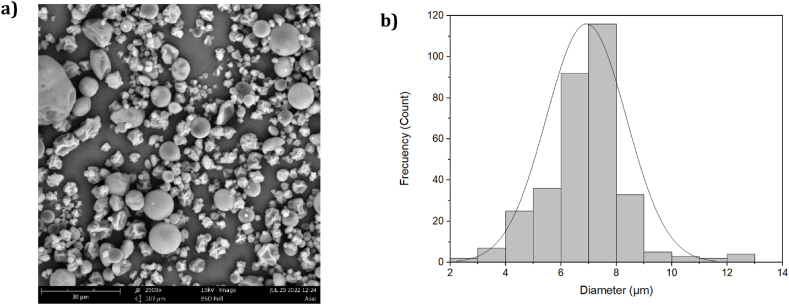
**a)** SEM image for the optimal powder of aҫai. **b)** Particle size distribution for optimized aҫai powder.

Fig. 4a shows the particles of the açaí pulp encapsulates obtained at optimal conditions. These presented various shapes, with some notably exhibiting a high level of sphericity, but most of them showed a variety of sizes (Fig. 4b), with diameters in a micrometer scale (most with a particle size of 6.91 **±** 1.45 μm) characteristic of materials obtained through spray-drying processes. However, certain particles displayed wrinkled surfaces, which could be attributed to the temperature and the drying rate. At higher temperatures, water evaporates more rapidly, resulting in particles with both smooth and hard surfaces. For this reason, a previous study [17] suggested conducting drying processes at temperatures above 170 °C. In their study involving açaí pulp, they reported that the particles dried at 138 °C exhibited more roughness compared to those dried at 202 °C, as illustrated in their micrograph.

## 4 Conclusions

This study was performed to optimize the spray drying process and evaluate its efficiency by focusing on the ascorbic acid content. Furthermore, it aimed to assess the effects of the drying process on the bioactive compounds and antioxidant activity of açaí pulp. The results demonstrated that the 2FI model successfully predicted 77 % of the variability in the ascorbic acid data. Similarly, all the response variables yielded results consistent with the desirable physical characteristics of the powder. The optimal factors for the açaí pulp spray-drying process were determined as follows: an inlet air temperature of 170 °C, 15 % maltodextrin, and a constant feed rate of 7.5 mL/min. The validation of the optimal conditions predicted by the model confirmed the predictive capacity of the models for the studied variables, achieving a desirability of 0.951. For the bioactive compounds and antioxidant activity, their high retentions indicated that spray drying successfully maintained the properties of açaí pulp, potentially benefiting both the quality of the final product and its potential health benefits. By encapsulating acai, which is naturally rich in phenolic compounds, flavonoids, anthocyanins, and ascorbic acid, these valuable bioactive constituents are shielded from environmental factors, ensuring their stability, extended shelf life, and protection against degradation. Furthermore, these findings provide valuable insights into the potential applications of açai pulp and its powder in various food and dietary products, considering the impacts on bioactive compound retention.

## CRediT authorship contribution statement

**Valentina Vargas:** Writing – review & editing, Writing – original draft, Methodology, Investigation, Data curation. **Sebastian Saldarriaga:** Writing – review & editing, Methodology, Data curation. **Francis S. Sánchez:** Writing – review & editing, Data curation, Conceptualization. **Liceth N. Cuellar:** Writing – review & editing, Methodology, Data curation, Conceptualization. **Gloria M. Paladines:** Writing – review & editing, Supervision, Methodology, Funding acquisition, Data curation, Conceptualization.

## Declaration of competing interest

The authors declare that they have no known competing financial interests or personal relationships that could have appeared to influence the work reported in this paper.
