# Supplementary material for: Effects of the spray-drying process using maltodextrin on bioactive compounds and antioxidant activity of the pulp of the tropical fruit açai (Euterpe oleracea Mart.)
Source: Heliyon. 2024 Jun 25;10(13):e33544. doi: 10.1016/j.heliyon.2024.e33544 (PMC11260920; doi:10.1016/j.heliyon.2024.e33544)
Supplement: Multimedia component 1 [file mmc1.docx]

**Table S1.** Proposed models for the adjustment the yield variable.

| **Source of variation** | **Sum of Squares** | **Mean Square** | | **F-value** | **p-value** |
| --- | --- | --- | --- | --- | --- |
| **Linear** | 195.80 | 97.90 | 1.04 | | 0.3921 |
| **2FI** | 62.84 | 62.84 | 0.6414 | | 0.4463 |
| **Quadratic** | 285.08 | 142.54 | 1.71 | | 0.2576 |

*Significant variables (p < 0,05)

**Table S2.** Proposed models for the adjustment the moisture variable.

| **Source of variation** | **Sum of Squares** | **Mean Square** | | **F-value** | **p-value** |
| --- | --- | --- | --- | --- | --- |
| **Linear** | 6.30 | 3.15 | 22.02 | | 0.0003* |
| **2FI** | 0.02 | 0.02 | 0.11 | | 0.7437 |
| **Quadratic** | 0.74 | 0.37 | 4.23 | | 0.6633 |

* Significant variables (p < 0,05)

**Table S3.** ANOVA for the model adjusted of moisture percentage

| **Source of variation** | **Sum of Squares** | **Mean Square** | **F-value** | **p-value** |  | **R^2^** | **Adjusted R^2^** | **Predicted R^2^** |
| --- | --- | --- | --- | --- | --- | --- | --- | --- |
| **Model** | 7.07 | 1.41 | 16.09 | 0.0020 | Significant | 0.9306 | 0.8728 | 0.7965 |
| **Residual** | 0.5269 | 0.0878 |  |  |  |  |  |  |
| **Lack of Fit** | 0.4543 | 0.1514 | 6.26 | 0.0832 | Not significant |  |  |  |

Significant model (p < 0,05); Not significant Lack of fit (p > 0,05); R^2^: coefficient of determination.

**Table S4.** Proposed models for the adjustment the water activity variable.

| **Source of variation** | **Sum of Squares** | **Mean Square** | | **F-value** | **p-value** |
| --- | --- | --- | --- | --- | --- |
| **Linear** | 0.0336 | 0.0168 | 31.78 | | <0.0001 |
| **2FI** | 0.0016 | 0.0016 | 4.05 | | 0.0790 |
| **Quadratic** | 0.0020 | 0.0010 | 5.51 | | 0.0438 |

* Significant variables (p < 0,05)

**Table S5.** ANOVA for the model adjusted of water activity.

| **Source of variation** | **Sum of Squares** | **Mean Square** | **F-value** | **p-value** |  | **R^2^** | **Adjusted R^2^** | **Predicted R^2^** |
| --- | --- | --- | --- | --- | --- | --- | --- | --- |
| **Model** | 0.0336 | 0.0168 | 31.78 | < 0.0001 | Significant | 0.8760 | 0.8484 | 0.6978 |
| **Residual** | 0.0048 | 0.0005 |  |  |  |  |  |  |
| **Lack of Fit** | 0.0043 | 0.0007 | 5.05 | 0.1058 | Not significant |  |  |  |

Significant model (p < 0,05); Not significant Lack of fit (p > 0,05); R^2^: coefficient of determination.

**Table S6.** Proposed models for the adjustment the WSI variable.

| **Source of variation** | **Sum of Squares** | **Mean Square** | | **F-value** | **p-value** |
| --- | --- | --- | --- | --- | --- |
| **Linear** | 10.60 | 5.30 | 30.37 | | <0.0001* |
| **2FI** | 0.1318 | 0.1318 | 0.7330 | | 0.4168 |
| **Quadratic** | 0.8950 | 0.4475 | 4.94 | | **0.0498*** |

* Significant variables (p < 0,05)

**Table S7.** ANOVA for the model adjusted of WSI.

| **Source of variation** | **Sum of Squares** | **Mean Square** | **F-value** | **p-value** |  | **R^2^** | **Adjusted R^2^** | **Predicted R^2^** |
| --- | --- | --- | --- | --- | --- | --- | --- | --- |
| **Model** | 11.63 | 2.33 | 25.66 | 0.0006 | Significant | 0.9553 | 0.9181 | 0.8552 |
| **Residual** | 0.5438 | 0.0906 |  |  |  |  |  |  |
| **Lack of Fit** | 0.4453 | 0.1484 | 4.52 | 0.1235 | Not significant |  |  |  |

Significant model (p < 0,05); Not significant Lack of fit (p > 0,05); R^2^: coefficient of determination.

**Table S8.** Proposed models for the adjustment the hygroscopicity variable.

| **Source of variation** | **Sum of Squares** | **Mean Square** | | **F-value** | **p-value** |
| --- | --- | --- | --- | --- | --- |
| **Linear** | 327.41 | 163.70 | 17.10 | | <0.0009* |
| **2FI** | 0.0029 | 0.0029 | 0.0003 | | 0.9873 |
| **Quadratic** | 19.09 | 9.54 | 0.8535 | | 0.4719 |

* Significant variables (p < 0,05)

**Table S9.** Proposed models for the adjustment the hygroscopicity variable

| **Source of variation** | **Sum of Squares** | **Mean Square** | **F-value** | **p-value** |  | **R^2^** | **Adjusted R^2^** | **Predicted R^2^** |
| --- | --- | --- | --- | --- | --- | --- | --- | --- |
| **Model** | 327.41 | 163.70 | 17.10 | 0.0009 | Significant | 0.7916 | 0.7453 | 0.7158 |
| **Residual** | 86.18 | 9.58 |  |  |  |  |  |  |
| **Lack of Fit** | 79.17 | 13.20 | 5.65 | 0.0918 | Not significant |  |  |  |

**Table S10.** Determination of ascorbic acid encapsulation efficiency in acai powder

| **Parameters** | **S** | **C** | **C1** | **AE** | **M** | **SD** |
| --- | --- | --- | --- | --- | --- | --- |
| B=170°C  A=15% | 0.5221 | 121.3991 | 232.5208 | 98.2786 | 98.9011 | 1.0026 |
|  | 0.5231 | 121.7409 | 232.7297 | 98.3669 |  |  |
|  | 0.5213 | 123.4072 | 236.7297 | 100.0576 |  |  |

B: inlet temperature; A: wall material proportion; S: weight of the powder (g); C: ascorbic acid concentration in powder (mg/L); C1: ascorbic acid concentration in powder (mg/100); AE: ascorbic acid encapsulation efficiency (%); M: mean (g/100 g); SD: standard deviation.

Ascorbic acid content in acai pulp powder was first obtained in mg/L and was expressed as mg/100 according to Equation (S1)

C1 (mg/100 g) = $\frac{C*0.01}{S}$ * 100 (S1)

Where C corresponds to ascorbic acid concentration in powder (mg/L); 0.01 corresponds to volume of water used to dissolve the powder (L) and S corresponds to weight of the powder (g).

Ascorbic acid encapsulation efficiency was calculated according to Equation (S2)

$\mathrm{AE}\left( \% \right)= \frac{C1*M_{e}}{C_{p}*M_{p}}*100$ (S2)

where C1 corresponds to ascorbic acid concentration in the powder (mg/100 g); C_p_ corresponds to ascorbic acid concentration in acai pulp (32 mg/100 g); M_e_ is the amount of powder obtained (40.7423 g) and M_p_ is the amount of pulp introduced in the feeding (301.23 g).

**Determination of** **Oxygen Radical Absorbance Capacity (ORAC)**

The antioxidant capacity, expressed as AUC, was calculated as:

*AUC=* 1 + $\frac{f_{1}}{f_{0}}$ + … + $\frac{f_{n}}{f_{0}}$

where AUC corresponds to area under the fluorescence decay curve; *f_0_* corresponds to initial fluorescence reading at 0 min; *f_n_* corresponds to fluorescence reading at time n. The concentration of the examined extracts, and Trolox were equalized to 0.1 mM.
